# A Vantage from Space Can Detect Earlier Drought Onset: An Approach Using Relative Humidity

**DOI:** 10.1038/srep08553

**Published:** 2015-02-25

**Authors:** Alireza Farahmand, Amir AghaKouchak, Joao Teixeira

**Affiliations:** 1 University of California, Irvine, California 92697, USA; 2 Jet Propulsion Laboratory, California Institute of Technology, Pasadena, California 91109, USA

## Abstract

Each year, droughts cause significant economic and agricultural losses across the world. The early warning and onset detection of drought is of particular importance for effective agriculture and water resource management. Previous studies show that the Standard Precipitation Index (SPI), a measure of precipitation deficit, detects drought onset earlier than other indicators. Here we show that satellite-based near surface air relative humidity data can further improve drought onset detection and early warning. This paper introduces the Standardized Relative Humidity Index (SRHI) based on the NASA Atmospheric Infrared Sounder (AIRS) observations. The results indicate that the SRHI typically detects the drought onset earlier than the SPI. While the AIRS mission was not originally designed for drought monitoring, we show that its relative humidity data offers a new and unique avenue for drought monitoring and early warning. We conclude that the early warning aspects of SRHI may have merit for integration into current drought monitoring systems.

Droughts can be described and assessed using different climatic variables such as precipitation, runoff and soil moisture^1^. For example, a meteorological drought is often described as a deficit in precipitation, an agricultural drought is expressed as a deficit in soil moisture, whereas a hydrological drought typically refers to below average surface or sub-surface water^2^. Given that droughts can be described relative to different variables, numerous drought indices have been developed based on one or more climatic variables^3^,^4^. For example, the Standardized Precipitation Index (SPI^5^,^6^) is widely used as an indicator of meteorological drought, while the Standardized Soil Moisture Index (SSI^7^) and soil moisture percentiles have been used for agricultural drought monitoring. A number of multivariate or multi-index indicators have also been developed such as the Joint Deficit Index^8^ and the Multivariate Standardized Drought Index^9^.

Drought monitoring indices show substantial variation in their ability to detect drought onset and termination^10^,^11^. Generally, precipitation measures detect drought onset earlier than other variables such as soil moisture and runoff^9^,^12^ because those variables have a delayed response to precipitation deficits. Consequently, the SPI detects the drought onset earlier than the SSI and soil moisture percentiles, and is thus more suitable for drought onset detection^12^. This study explores whether even earlier drought onset detection can be accomplished by factoring in the meteorological variables that influence precipitation. It is hypothesized that near surface air relative humidity (hereafter, relative humidity) can detect drought onset earlier than indications provided by precipitation signals. Relative humidity is an important climate variable defined as the ratio of air vapor pressure to the saturated vapor pressure. Precipitation and relative humidity are related to each other in the sense that precipitation is not expected at low relative humidity^13^.

Limitations in ground-based observations^14^ make satellite observations important for monitoring drought-related variables^15^,^16^,^17^,^18^. These limitations include uneven distribution of ground-based observations, temporal inconsistencies and spatial inhomogeneity in the records, and lack of observations in remote regions^14^. The Evaporative Stress Index^15^, the Global Terrestrial Drought Severity Index^3^, and the Global Integrated Drought Monitoring and Prediction System (GIDMaPS^19^) all highlight the value of remote sensing observations for monitoring drought.

We show that drought onset can be detected by standardizing relative humidity data via the relative humidity from the Atmospheric Infrared Sounder (AIRS^20^) satellite mission. Importantly, this detection can be earlier than that indicated by measures of precipitation and soil moisture. The mission's Version 6 data sets are obtained from two instruments: The Atmospheric Infrared Sounder (AIRS) and the Advanced Microwave Sounding Unit (AMSU). AIRS is an infrared spectrometer and radiometer with 2378 spectral channels ranging 3.7–15 *µm*. AMSU is a 15-channel microwave radiometer covering 23 to 89 GHz^20^,^21^. AIRS's monthly surface relative humidity (over equilibrium phase) is utilized for drought onset detection (Version 6, Level 3 data). The relative humidity data are available globally at a 1° spatial resolution (2002-present). AIRS products are available from ascending and descending tracks, which refer to the direction of movement of the sub-satellite point in the satellite track. We used the descending AIRS data, in which the direction of the movement is from Northern Hemisphere to Southern Hemisphere, with an equatorial crossing time of 1:30 AM local time^22^. To evaluate drought detection using relative humidity, the SPI and SSI data from GIDMaPS^19^ are used as additional indicators.

Typically, drought onset assessment is based on a certain drought threshold. In this study, the D0-Drought (Abnormally Dry^23^) condition is used as the drought onset threshold, which corresponds to a drought event with an approximately 30% probability of occurrence. As an example, the global SPI, SSI and SRHI maps for August 2010 are presented in Figure 1a, Figure 1b, and Figure 1c respectively. As shown, all three indices captured the Russian drought. This event and its accompanying heat waves resulted in thousands of casualties and significant economic losses in Russia and eastern Europe^24^. The Amazon drought was another major event in 2010, which led to substantial water level decreases in major Amazon tributaries^25^. At the other extreme, August 2010 was abnormally wet in eastern Australia. These patterns of wet and dry conditions are reflected on all three indices. Overall, Figure 1 illustrates that SRHI is consistent with SPI at wet and dry conditions, though there are discrepancies primarily around neutral condition (SPI and SRHI around 0).

To analyze drought early detection, we investigated time series of the SPI, SSI and SRHI over three major drought events: the 2010 Russian drought (Figure 2a), the 2010–2011 Texas-Mexico drought (Figure 2b), and the 2012 United States drought (Figure 2c). As we show, in the 2010 Russian drought, the SRHI indicates the onset nearly two months before both the SPI (Figure 2a - compare indices relative to the D0 threshold identified by the green horizontal line). Note that for a more severe drought condition (e.g., a lower threshold of -1), the SRHI detects the drought's onset even earlier.

The same drought indicators over one location in the Texas-Mexico Drought are displayed in Figure 2b. This series confirms that the SRHI identifies onset of this drought earlier than the other indicators. Finally, Figure 2c shows the SRHI, SPI and SSI over a specific location in an area affected by the 2012 United States drought. The 2012 drought was one of the most devastating events in the modern times and led to billions of U.S. dollars in economic damage. This event in particular affected crop development and early detection could have reduced agricultural losses^26^. As shown in Figure 2c, the SRHI detects the onset of this drought 3 to 4 months earlier than SPI. Such early detection in the growing season could potentially reduce the effects of droughts on agriculture and society^26^.

In the top three panels in Figure 2, the SRHI is the earliest drought detector, followed by SPI and SSI. The results are consistent with previous studies indicating that SPI detects the drought onset earlier than SSI. However, the results also show that remotely sensed relative humidity can be used for even earlier drought detection. While the SRHI does show the potential to advance drought early detection, in some cases it may not detect the drought onset earlier than the SPI (e.g., see Figure 2d where the SRHI detects the drought onset later than the SPI). Nonetheless, in all four cases shown in Figure 2, the SRHI is consistent with the SPI and SSI on showing the drought signal.

To assess the potential capability of AIRS relative humidity data in drought detection, we statistically evaluated the global SRHI values against SPI during 2002–2013 period. Figure 3a presents the probability of drought detection. Figure 3b and Figure 3c shows the false drought ratio and missed drought ratio respectively. Figure 3a shows the fraction of the reference data (i.e., negative SPI) identified correctly by the SRHI (perfect score = 1), whereas Figure 3b describes the fraction of drought events identified by SRHI, but not confirmed with the SPI (perfect score = 0)^27^. Figure 3c displays the fraction of drought events identified by SPI, but missed in SRHI (perfect score = 0). Given that there are limited number of droughts in each pixel during 2002–2013, the global statistics is derived for each 10 × 10 pixels to ensure the statistics is reliable.

An important question is in cases where a drought was detected by both SRHI and SPI, what fraction of events is detected earlier by SRHI. To answer this question, the drought onset based on SRHI (*DO_SRHI_*) is evaluated against that of SPI (*DO_SPI_*). To avoid unreliable statistics, only drought events longer than three months have been considered. Figure 4a shows the probability of drought detection (i.e., fraction of detected drought) when *DO_SRHI_* ≤ *DO_SPI_*. As shown, in most parts of the globe this fraction ranges between 0.5 to 0.8, with the global average being approximately 0.6 (i.e., 60% of all events). Figure 4b displays the mean lead time for each pixel based on SRHI relative to SPI. The figure indicates that the mean lead time ranges between 1 to 3 months with the global average being approximately 1.9 months. The results presented in Figures 3 and 4 do not show a strong regional/geographical pattern. This indicates that in most parts of the world the SRHI, combined with other indicators, can potentially improve early drought detection.

The SRHI's main limitation is the relatively short length of record (2002-present). However, there are other data sets with similar length of record that provide valuable drought information (e.g., GRACE observations, and Evaporative Stress Index data). SRHI can provide valuable information on current conditions but it cannot be used to put an extreme event in historical perspective. In a recent study, a Bayesian algorithm is proposed for combining multiple precipitation data to create a long-term climate data record^16^. Similar algorithms could be used to extend AIRS relative humidity data by combining it with reanalysis data sets (e.g., Modern-Era Retrospective Analysis for Research and Applications^28^). Efforts are underway to create a long-term and real-time relative humidity data set for drought monitoring and assessment. On the other hand, the current resolution (1°) of the relative humidity data only allows regional to continental scale drought assessment.

We believe that drought monitoring should be based on multiple sources of information. The proposed SRHI is not meant to replace the currently available indicators. Rather, it should be used alongside other drought indicators. This paper does not claim that SRHI alone is always sufficient for early drought detection. In fact, previous studies highlight the limitations of individual drought indicators^11^,^29^. Having an additional source of information based on relative humidity can improve our understanding of the drought onset and development. Precipitation, relative humidity, water vapor, temperature and vapor pressure deficit are closely related. For this reason, satellite-based temperature, water vapor and vapor pressure deficit information can also be explored for further improving drought onset detection. Moreover, several studies argue that statistical seasonal drought prediction is very sensitive to the initial meteorological and land-surface conditions^30^,^31^,^32^. Early drought detection can potentially lead to improvements in statistical seasonal drought prediction by providing additional information on the initial meteorological conditions. This issue, however, requires more in-depth research in the future.

Drought early onset detection is fundamental to local and regional mitigation plans, especially in the agriculture and water resources sectors. A water manager may need drought information months in advance for water resource planning, while for a farmer even few weeks of lead time is very important. Early detection, even by few weeks/months, allows farmers and local agencies to take adaptive measures that include purchasing less fertilizer and increasing insurance coverage, especially before or early in the growing season. The results highlight that the AIRS near surface air relative humidity data can potentially be used for drought early warning if integrated into currently available systems such as the U.S. Drought Monitor^23^ or GIDMaPS^19^.

## Methods

Standardized drought indices are often derived by normalization after fitting a parametric distribution function to the data^5^. However, a single parametric distribution may not fit data from different climatic regions^33^. In this study, the Standardized Relative Humidity Index (SRHI) is proposed using a non-parametric standardization approach.

First, the empirical probabilities of the AIRS relative humidity data are computed for each grid, using the empirical Gringorten plotting position^34^: 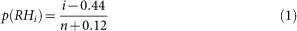
Where *i* is the rank of relative humidity (RH) data from the smallest, and *n* is the sample size. In this study, an empirical approach is used to avoid any assumption on the underlying distribution function of relative humidity data across space^35^. The empirical probabilities of relative humidity (*p*(*RH_i_*)) is then standardized as: 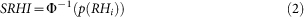
Where Φ^−1^ is the inverse standard normal distribution function with the mean of zero and standard deviation of one. Here, the standardization is based on the following approximation^36^,^37^: 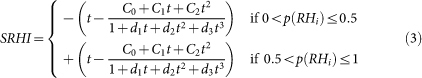
where *c*_0_ = 2.515517; *c*_1_ = 0.802583; *c*_2_ = 0.010328; *d*_1_ = 1.432788; *d*_2_ = 0.189269; *d*_3_ = 0.001308; and 
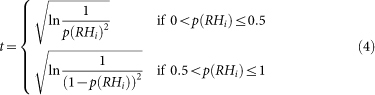
A negative SRHI is an indication of below average (climatology) relative humidity, and is proposed as a measure of dryness. One attractive feature of SRHI is that, similar to SPI, it can be derived for different time-scales (e.g., 1-, 3-, 6-month SRHI). For consistency and cross-comparison, the three indicators (SRHI, SPI and SSI) are computed using the same non-parametric approach and for a 3-month time scale.

## Figures and Tables

**Figure 1:**
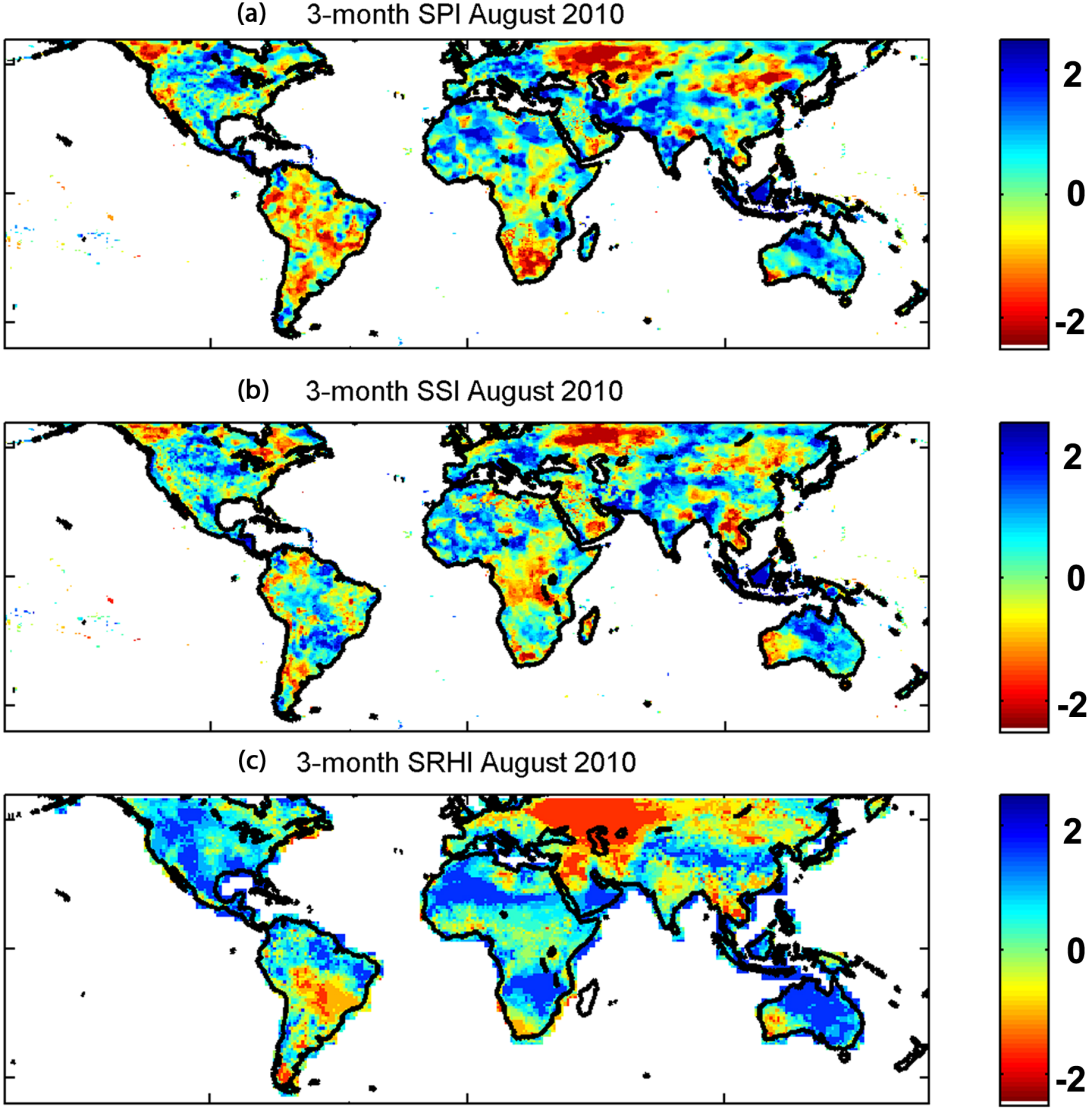
Global Standardized Precipitation Index (SPI), Standardized Soil Moisture Index (SSI), and Standardized Relative Humidity Index (SRHI) for August 2010. This map was generated using MATLAB.

**Figure 2:**
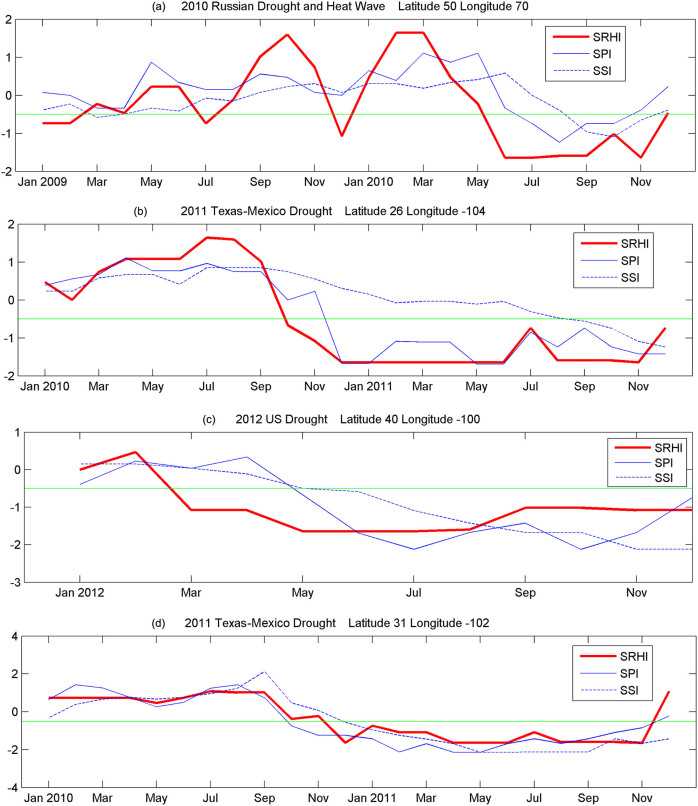
Time series of 3-month SPI, SSI and SRHI for several locations in areas affected by the 2010 Russian drought, 2010-2011 Texas-Mexico drought, and 2012 United States drought.

**Figure 3:**
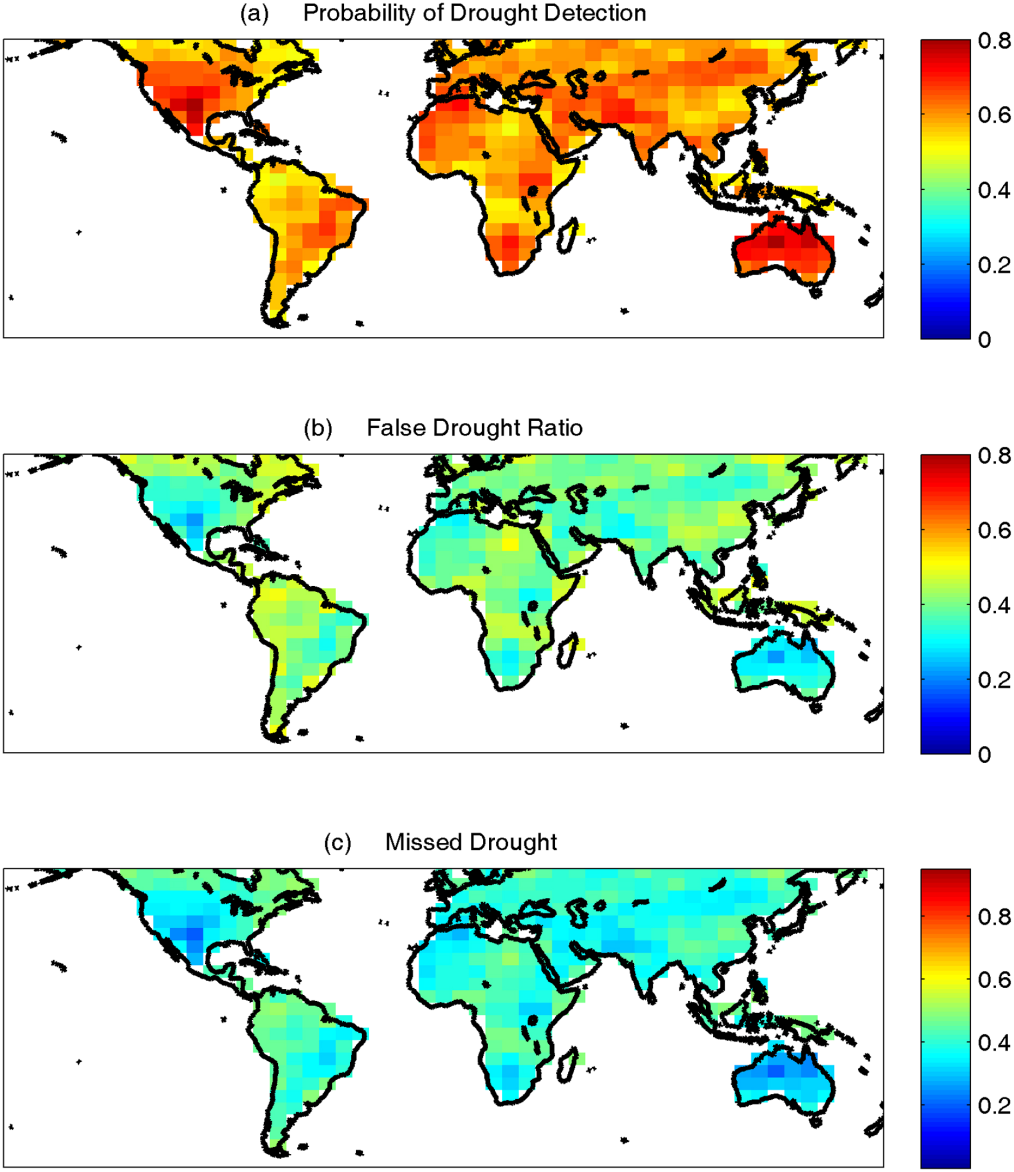
Probability of drought detection (a), false drought ratio (b), and missed drought ratio (v) for the SRHI relative to SPI. This map was generated using MATLAB.

**Figure 4:**
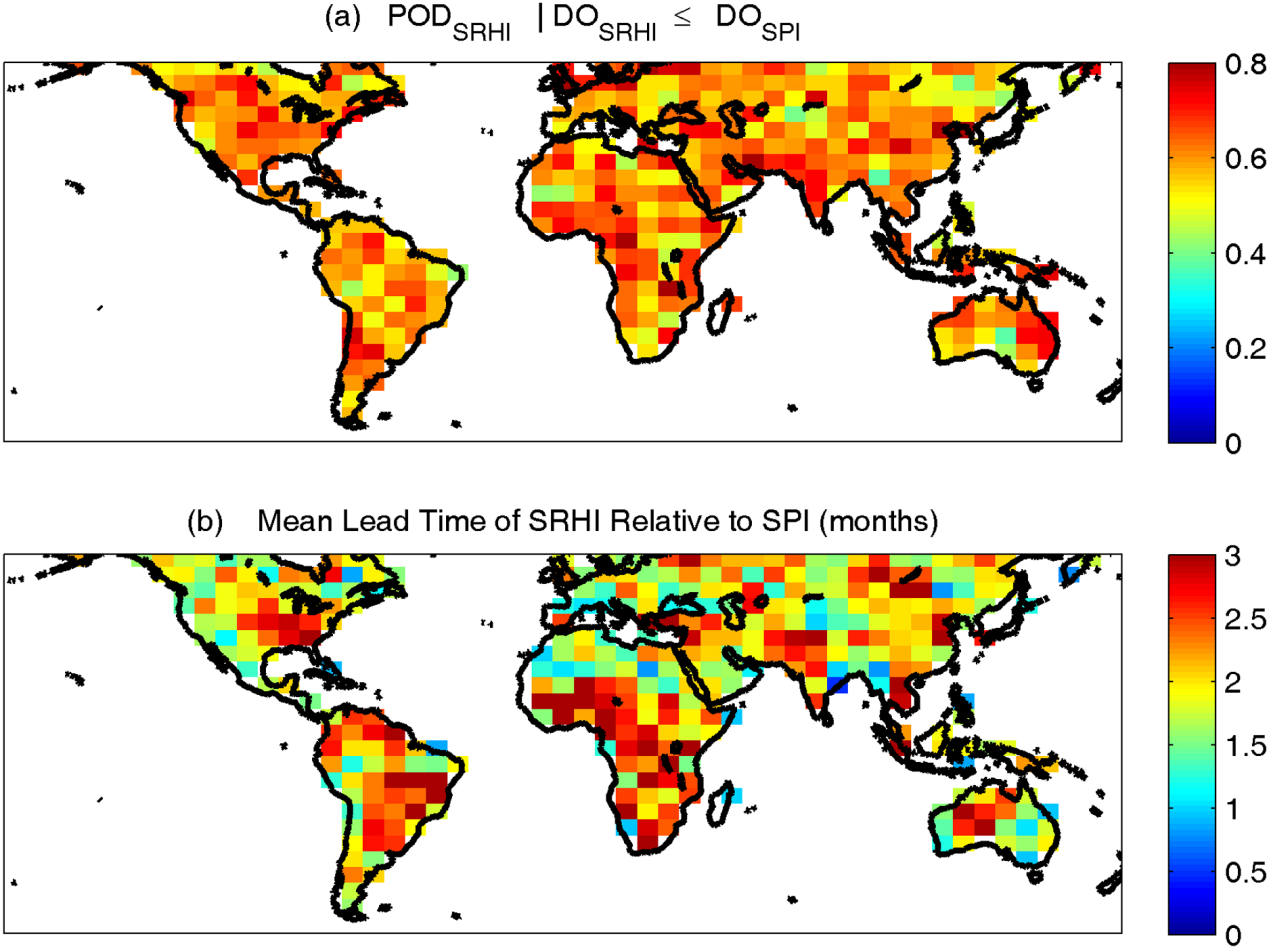
Probability of drought detection (i.e., fraction of detected drought) when Drought Onset (DO) based on SRHI is less or equal to that of SPI (*DO_SRHI_* ≤ *DO_SPI_*) (a), mean lead time based on SRHI relative to SPI (months) (b). This map was generated using MATLAB.
